# Precision and recall oncology: combining multiple gene mutations for improved identification of drug-sensitive tumours

**DOI:** 10.18632/oncotarget.20923

**Published:** 2017-09-15

**Authors:** Stefan Naulaerts, Cuong C. Dang, Pedro J. Ballester

**Affiliations:** ^1^ Computational Biology and Drug Design, Cancer Research Center of Marseille, INSERM U1068, Marseille, France; ^2^ Institut Paoli-Calmettes, Marseille, France; ^3^ Aix-Marseille Université, Marseille, France; ^4^ CNRS UMR7258, Marseille, France; ^5^ Faculty of Information Technology, VNU University of Engineering and Technology, Hanoi, Vietnam

**Keywords:** biomarker discovery, machine learning, drug sensitivity, genomics, cancer

## Abstract

Cancer drug therapies are only effective in a small proportion of patients. To make things worse, our ability to identify these responsive patients before administering a treatment is generally very limited. The recent arrival of large-scale pharmacogenomic data sets, which measure the sensitivity of molecularly profiled cancer cell lines to a panel of drugs, has boosted research on the discovery of drug sensitivity markers. However, no systematic comparison of widely-used single-gene markers with multi-gene machine-learning markers exploiting genomic data has been so far conducted. We therefore assessed the performance offered by these two types of models in discriminating between sensitive and resistant cell lines to a given drug. This was carried out for each of 127 considered drugs using genomic data characterising the cell lines. We found that the proportion of cell lines predicted to be sensitive that are actually sensitive (precision) varies strongly with the drug and type of model used. Furthermore, the proportion of sensitive cell lines that are correctly predicted as sensitive (recall) of the best single-gene marker was lower than that of the multi-gene marker in 118 of the 127 tested drugs. We conclude that single-gene markers are only able to identify those drug-sensitive cell lines with the considered actionable mutation, unlike multi-gene markers that can in principle combine multiple gene mutations to identify additional sensitive cell lines. We also found that cell line sensitivities to some drugs (e.g. Temsirolimus, 17-AAG or Methotrexate) are better predicted by these machine-learning models.

## INTRODUCTION

The analysis of tumour DNA has been investigated as a way to personalise cancer therapies for quite some time [1]. This analysis usually leads to the detection of somatic mutations, such as a specific single-nucleotide variant (SNV) or copy-number alteration (CNA), on oncogenes and tumour suppressor genes. Somatic mutations can affect the abundance and function of gene products driving tumour growth at the molecular level. These mutations can hence influence disease outcome and/or response to a given drug [2]. Genetic information has thus been found valuable for aiding the selection of effective treatment by relating the molecular profile of tumours to their observed sensitivity to drugs [3, 4].

Preclinical research on the identification of drug-gene associations that can be used as single-gene markers of drug response is often carried out on human cancer tumour-derived cell lines [5–8]. Cell lines permit relatively quick and cheap large-scale *in vitro* experiments, which are generally not feasible on more accurate *ex vivo* or *in vivo* disease models [9, 10]. Here the somatic mutations of the untreated cell line are determined first. The viability of cells is thereafter measured to assess their intrinsic sensitivity or resistance to the tested drug. Lastly, the resulting *in vitro* pharmacogenomic data is analysed to establish which drug-gene associations are statistically significant and hence proposed as single-gene markers. In addition to single-gene marker discovery [6, 8, 11], such data sets have also been used for the development of multi-variate models of cell sensitivity to drugs of various types (pharmacogenomics [12–14], pharmacotranscriptomics [15–19], QSAR [20, 21]) and their applications (drug repositioning [20, 22], molecular target identification [22–24]). These *in silico* models are built with algorithms that learn from data, which are studied in the field of machine learning [25]. A common type of machine-learning algorithms generates classification models, also known as classifiers, which are often used to learn to group cell lines into two categories (sensitive or resistant to a drug). Pharmacogenomic data from the Genomics of Drug Sensitivity in Cancer (GDSC) [26] constitute one of the most comprehensive resources for methodology research on the identification of optimal genomic markers of cancer drug sensitivity (e.g. NCI-60 drugs are tested against only 59 unique cell lines [5] and the CCLE assembled a larger collection of cell lines than GDSC but tested a smaller subset of cell lines per drug [7]).

Predictive models based on GDSC data have been mostly restricted to single-gene markers of drug sensitivity [6]. However, multi-gene models have been used for the related purpose of estimating the importance of somatic mutations for cell line sensitivity to each drug [6]. By contrast, we subsequently investigated the performance of multi-gene machine-learning models exploiting GDSC data on the prediction of cell sensitivity to drugs [12]. As in other efforts [7, 13, 14], we did not investigate how well machine-learning models perform compared to single-gene markers across GDSC drugs. It is now clear that such comparative analysis is essential to understand the benefits provided by modelling multiple gene alterations. Beyond this research area, multi-variate machine-learning models are also starting to be advocated for genomic-based prediction of other complex phenotypic traits [27].

In practice, models based on one feature (single-gene markers) can outperform models based on more than one feature (multi-variate classifiers). This is partially due to cell lines being often characterised by sparsely-valued binary features (i.e. features that are only present in a small fraction of the cell lines), which poses a challenge to classifiers acting on a high-dimensional feature space in that few differences between cell lines are available to support their effective discrimination. This leads to the following question: for which drugs are multivariate markers more predictive of cell line sensitivity than univariate markers? A recent study has finally investigated this question using large-scale GDSC data [8]. In brief, LOBICO logic modelling was used to build classifiers of predetermined complexity for each drug, followed by retaining the logic model with the best cross-validated performance. Due to the computational expense of seeking an optimal solution, these classifiers could only incorporate up to four binary features (e.g. whether four genes were mutated or not in the considered cell line). While the training procedure of LOBICO mathematically guarantees that the retained model will be the best among those based on binary features for a given complexity [28], it is important to note that better models can exist with higher complexities (e.g. those using more than four features), broader exploration of feature space (e.g. whole-exome variants), using continuous features (e.g. gene expression levels) or by simply using different sets of binary features (e.g. whether a particular point mutation is present in the gene instead of whether this gene has any mutation). A second limitation is that machine-learning models were only used to establish which molecular profiles were more informative on average across all drugs. Hence, the performances of these models were not compared against those of single-gene markers (this was only done with logic models). Third, both logic model selection and its classification performance measurement were carried using the same data folds in the adopted cross-validation procedure. Therefore, these cross-validated results provide an overoptimistic performance assessment of the selected model and result in not selecting models that would perform better on truly independent test sets, as demonstrated elsewhere [29–31].

Here we study the performance of machine learning exploiting all available gene mutations (instead of being limited to models using up to four mutations). This analysis is conducted as it would be done in practice by selecting the best single-gene marker and the best multi-gene model of the considered drug on a training set representing the data available at the time of model selection. We assess thereafter both models in an unbiased manner using a time-stamped independent test set, i.e. data that was released after training data and not used for model building or model selection. The advantages of using a time-stamped data partition are that this mimics a blind test: the experimenters avoid selecting a particular partition (perhaps best performing), we do not use the same data for both the selection and assessment of models (thus avoiding performance overestimation) and the negative impact of time-dependent batch effects is considered (e.g. drug sensitivities can diverge if different batches of fetal bovine serum are used in the cell cultures [32]).

Our focus is on somatically mutated genes for several reasons. First, it has been demonstrated that the performance of pan-cancer markers of drug sensitivity on an independent test set is most relevant to help to stratify patients for basket trials [33], where patients are included if their tumours harbour a particular gene mutation regardless of cancer subtype. Second, drug sensitivity predictors integrating data from multiple molecular profiling technologies are less amenable for clinical implementation due to much higher requirements in cost, time and resources per patient [34, 35]. Therefore, there is a practical need to understand how machine-learning models can improve the performance of single-gene markers in the context of a given profiling technology. While this issue has recently been investigated for gene expression profiles [31], it is currently unknown for which drugs combining multiple gene mutations via machine learning can outperform standard single-gene markers. Due to their relevance for both clinical and research contexts, we present here a full comparison of the predictive value of single-gene and multi-gene genomic markers.

## RESULTS

### How to compare single-gene and multi-gene markers of drug response

A drug-gene association or single-gene marker is effectively a classifier that uses a single independent variable or feature (the considered somatic DNA mutation). External validation of single-gene markers is unusual, as the validity of the drug-gene association is commonly established by showing that a statistically significant p-value is attained. In this sense, machine learning represents a different culture [36], where the validity of the predictor can only be claimed when its prediction is better than random on a test set independent of the employed training set.

As detailed in the Methods section, two non-overlapping data sets are generated per drug. For clarity, we will briefly summarize the datasets here. First, the training set containing the cell lines tested at the time of the first data release along with their IC_50_s for the drug and 71 binary features corresponding to the presence or absence of the considered gene alterations. Second, the test set contains the new cell lines tested for that drug and released after data in the training set. Training and test sets are hence non-overlapping by construction. We used the median logIC_50_ in μM units of all cell lines in the training set to define the sensitivity threshold for both the training set and the test set, which is required to assign the class labels (sensitive, resistant) to each drug-cell pair.

**Figure 1:**
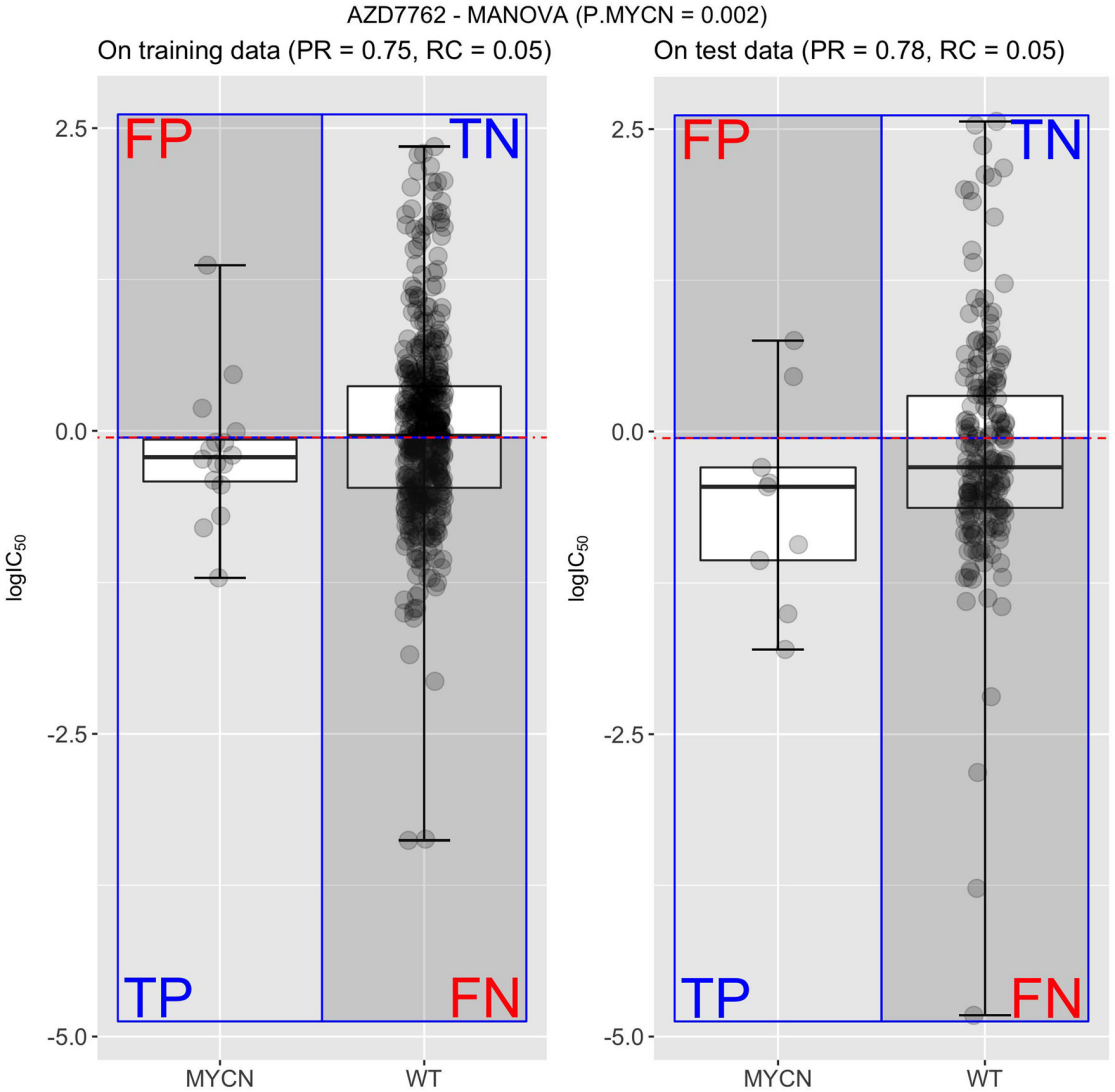
Measuring the performance of the most significant single-gene marker for the drug AZD7762 Using all the cell lines originally tested with this drug (the training set), MYCN-mutated cell lines were found to have increased sensitivity to AZD7762 (P=0.002)^6^. The median logIC50 in μM units of all cell lines in this training set (left) defines the sensitivity threshold (horizontal red line) for both training and test sets. Thus, this single-gene classifier predicts that cell lines harbouring the genetic aberration (mutated MYCN with boxplot named MYCN for short), tend to be more sensitive to AZD7762 (logIC50 below threshold) than cell lines that are wild-type (WT) with respect to the MYCN gene (boxplot marked as WT for short). This classifier achieves a high precision (PR=0.75) on the training set because only 25% of the cell lines that are predicted to be sensitive are actually resistant (false positives; FP). However, it obtains a very low recall (RC=0.05) as the vast majority of sensitive cell lines are WT with respect to MYCN and hence are incorrectly predicted to be resistant (i.e. false negatives; FN). A second scatter plot with two boxplots (right) shows the classification performance of the AZD7762-MYCN marker on the test set. It is similar to the performance obtained on the training set, which evidences the robustness of this particular marker.

For each drug, we then generated its confusion matrix, which displays four distinct performance metrics: the numbers of true positives (TP), true negatives (TN), false positives (FP) or false negatives (FN). An example is shown in Figure 1, where these four categories are calculated for the association between the drug AZD7762 and its most significant single-gene marker (MYCN, p-value = 0.002). Note its high precision, but low recall (also known as sensitivity). Indeed, this single-gene classifier achieves a high precision (PR=0.75) on the training set (left-hand side) because only 25% of the cell lines that are predicted to be sensitive are actually resistant (false positives). By contrast, it obtains a very low recall (RC=0.05) as the vast majority of sensitive cell lines are wild-type (WT) with respect to MYCN and hence are incorrectly predicted to be resistant (i.e. false negatives). Performance on the test set is shown on the right and in this case is similar to that in the training set, which demonstrates the robustness of this particular single-gene marker. Based on the confusion matrix, we subsequently summarized the discrimination offered by a classifier with the Matthews Correlation Coefficient (MCC), Precision (PR), Recall (RC) and F-score (F1). These performance metrics are fully detailed in the Methods section.

To allow a direct comparison with the best single-gene marker of each drug, we built a Random Forest (RF) classification model [37] for each drug using the same training data set as the corresponding single-gene marker. We selected RF as this machine-learning technique has been shown to be suitable for high-dimensional problems involving genomic data [38] and also works well on GDSC data [12], although we make no claim about the optimality of this choice. Each RF model was built with 1000 trees, with its m_try_ control parameter being selected by 10-fold cross-validation as specified in the Methods section. The RF classifier of each drug is evaluated on the corresponding test set. To remind the reader about this stringent evaluation of models, we will sometimes highlight that a given performance metric was calculated on the independent test set by explicitly stating so (e.g. referring to “test set precision” instead of just “precision”). The next sections present a comparative assessment of both single-gene and multi-gene markers.

### Precision varies strongly depending on whether a single-gene or multi-gene marker is used

**Figure 2:**
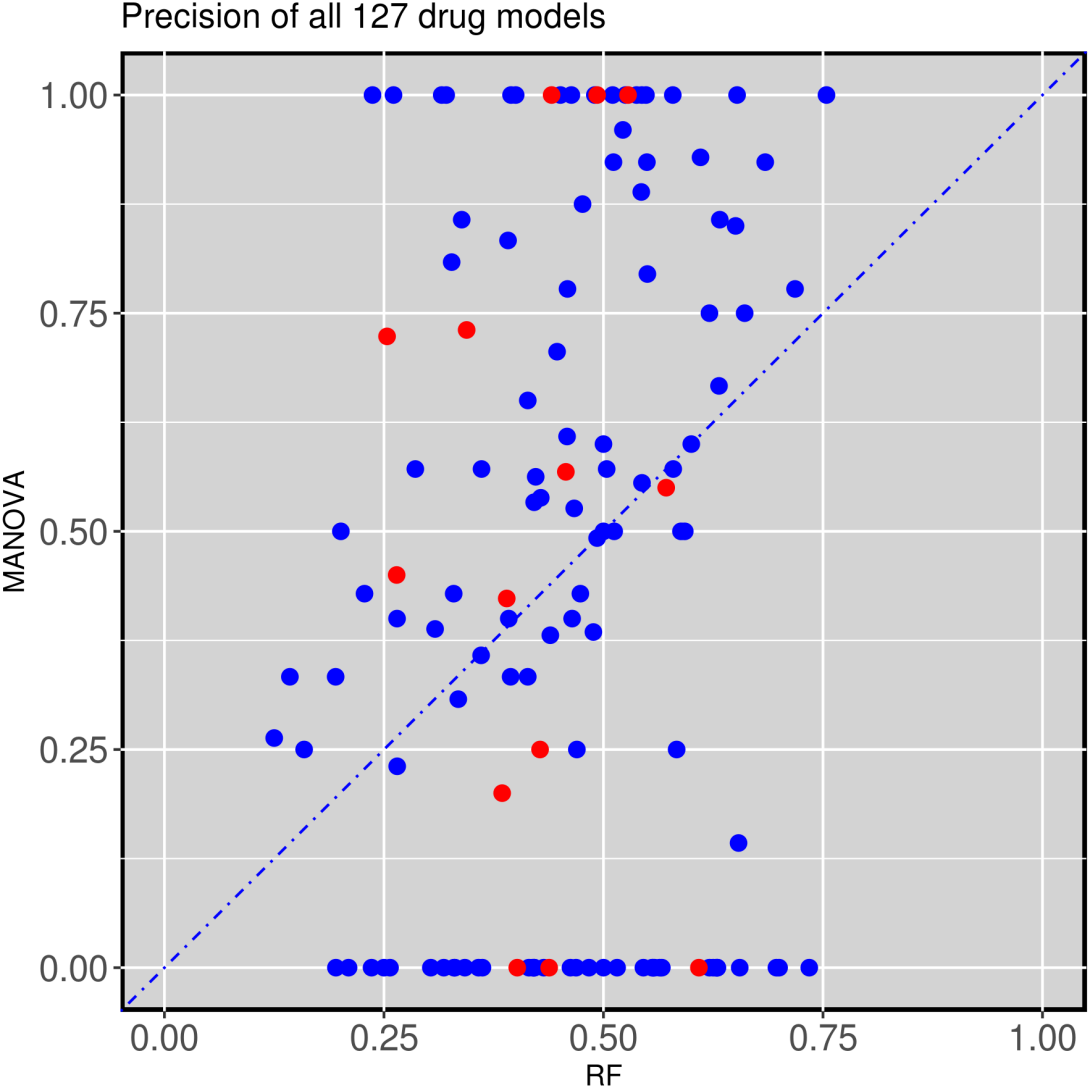
Test set precision of MANOVA single-gene markers versus RF multi-gene markers across 127 drugs 62 drugs obtain better precision with the RF classifiers combining the 71 gene alterations, while 65 of the single-gene markers have higher precision. Cytotoxic drugs are red-coloured and targeted drugs are blue-coloured following the annotation of the original GDSC study.

Figure 2 compares the test set precision of single-gene MANOVA markers versus multi-gene RF across the 127 drugs. In this context, precision is the proportion of cell lines predicted to be sensitive that are actually sensitive (for single-gene markers, precision translates to the response rate in cell lines with the actionable mutation). A great variability is observed depending on which type of classifier is used as a marker for a given drug: 65 drugs are predicted with higher precision by a single gene alteration, whereas 62 drugs have higher precision when predicted by combining multiple gene alterations via RF (Supplementary Table 1). This is often true for MANOVA markers based on a relatively rare mutation, as often no test cell line harbours such gene mutation, in which case test set precision is zero. We further distinguished between cytotoxic and targeted drugs. Targeted drugs hit a target or targets that are particularly important for tumoral cells, whereas cytotoxic drugs affect basic cellular machinery and thus typically elicit a broader effect reaching both tumoral and non-tumoral cells alike [39]. No evident trend was found in the precision of the classifier depending on whether this was constructed for a targeted or cytotoxic drug (Figure 2).

### External validations highlight the limitations of single-gene markers

**Figure 3:**
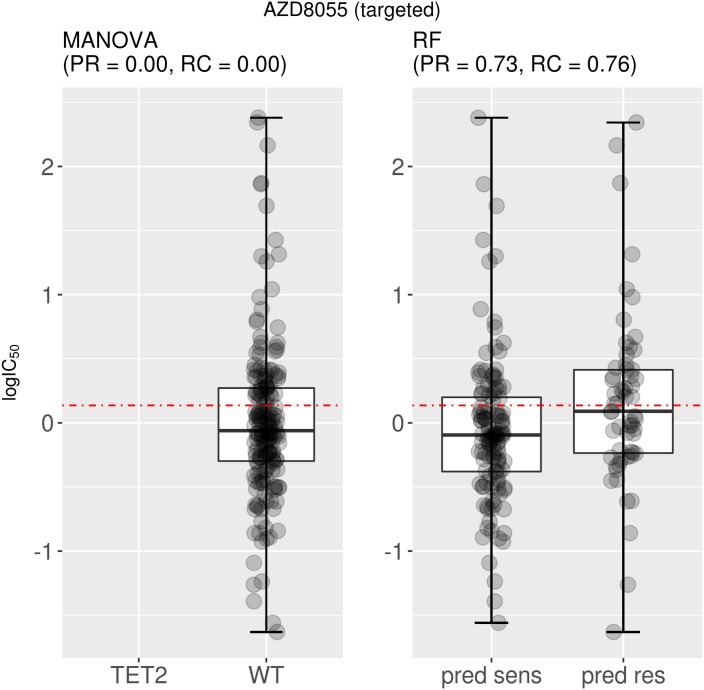
Best MANOVA single-gene marker versus RF multi-gene marker for AZD8055 Although the correlation is clear on the training set, the TET2 marker turned out to be of no use for the test set, as none of the test cell lines harbour mutations in the TET2 gene (no boxplot on the left). By contrast, the multi-gene marker is highly precise in identifying sensitive cell lines (PR=0.73) and does so with a high recall (RC=0.76). Note that the boxplot named “pred sens” shows the sensitivities of test set cell lines predicted sensitive, whereas the boxplot labeled “pred res” displays those for the test set cell lines predicted resistant.

Next we analyse two examples of drugs for which the test set precision obtained by the multi-gene marker is similar or higher than that of the single-gene markers. This analysis uncovered two severe issues with single-gene markers. The first issue is that the single-gene marker is often not found in the test tumours. For example, the drug AZD8055 targets both the mTORC1 and mTORC2 kinase complexes in the mTOR signaling cascade (Reactome: R-HSA-165159) and had as most significant single-gene marker TET2. This AZD8055-TET2 relationship was characterized by a p-value of 3·10^-5^. However, test set cell lines do not include any TET2 mutations, voiding the use of this marker entirely (Figure 3). By contrast, the multi-gene RF was capable of predictive sensitive cell lines in the test set with precision and recall rates exceeding 70% (Figure 3). Figure 2 can be revisited to place both precision values for AZD8055 in the context of those for the rest of drugs (all performance results are available in Supplementary Table 1). As such, single-gene markers suffer from being unable to predict at least a portion of the sensitive population. It is therefore mandatory that additional genes are considered to assist the preferred single-gene marker to accommodate for this lost part of the sensitive population. Consequently, significance of p-values cannot be the sole requirement when identifying biomarkers. We found that this problem is particularly acute in cases where the MANOVA marker is based on a relatively rare mutation. For example, the actionable mutation was not present in any of the test set cell lines for 20.5% of the best single-gene markers (26 of the 127 drugs) and therefore these held no predictive value for the test set (zero precision). The identities of these 26 drugs are available in Supplementary Table 1 along with all the results for both types of markers on each data set.

**Figure 4:**
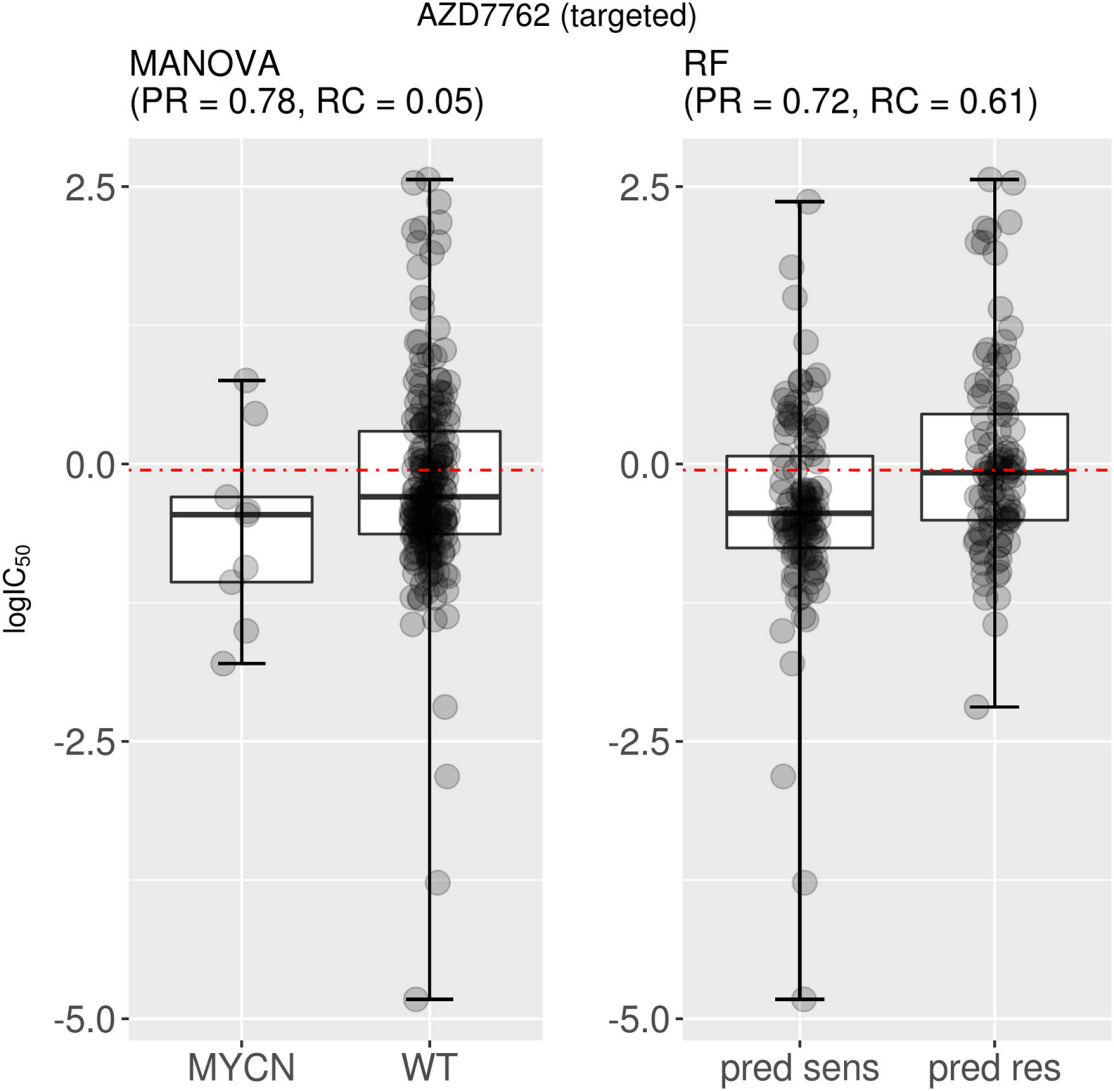
Best MANOVA single-gene marker versus RF multi-gene marker for AZD7762 This targeted drug has similar precision for the single-gene MYCN mutation marker compared to the multi-gene marker (PR=0.78 vs PR=0.72). However, the recall is vastly different between classifier types (MANOVA RC=0.05; RF RC=0.61). Taken together, these results indicate that the multi-gene classifier predicts cell line sensitivity to AZD7762 substantially better than its best single-gene marker. Note the following abbreviations: “pred sens” (predicted sensitive) and “pred res” (predicted resistant).

The second issue is that the single-gene marker can have very low recall. For instance, AZD7762 is a drug that primarily targets CHEK1 and CHEK2 in the G2/M DNA damage checkpoint pathway (Reactome: R-HSA-69473), which is essential for the inactivation of the Cyclin B:cdk1 complex when triggered by DNA damage. The best single-gene response marker for AZD7762 is MYCN, which is significantly associated with this drug (P= 0.002 on the training set). The two boxplots in Figure 4 represent the test set performance of both the MANOVA and the RF for a targeted drug (AZD7762). As can be seen in Figure 4, 78% of MYCN-mutant cell lines are predicted as sensitive to this drug according to the single-gene model, whereas this response rate is 72% when combining the 71 gene alterations. Although a similar precision was reported, the RF model was found to have a strongly improved recall (0.61) compared to that of the single-gene marker (0.05).

### Multi-gene markers generally achieve much higher recall than single-gene markers

**Figure 5:**
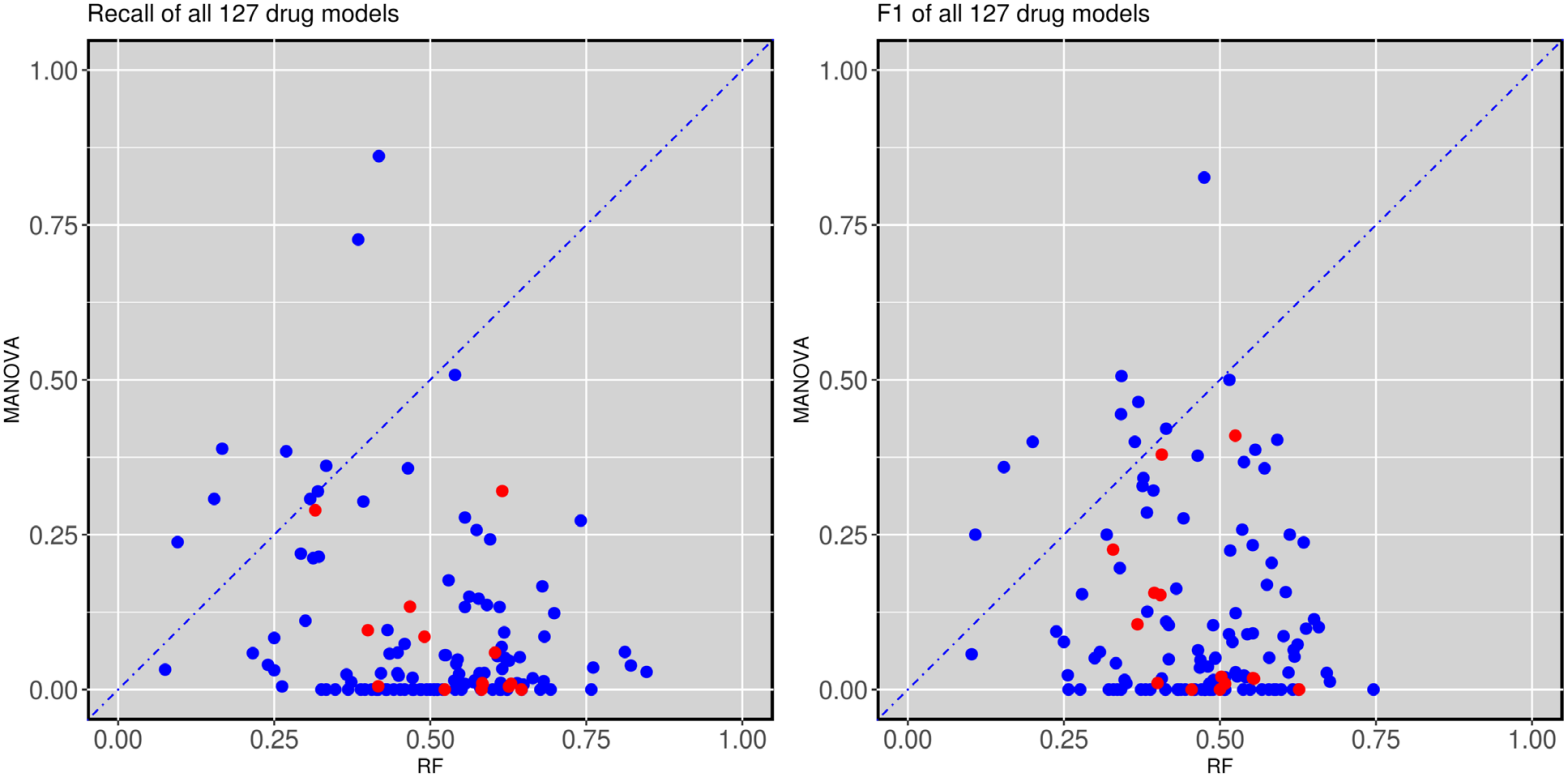
Test set recall and F-scores of single-gene versus multi-gene markers across the 127 drugs **(left)** Multi-gene markers achieve a higher recall than single-gene markers in 118 of the 127 drugs. **(right)** Similarly, multi-gene markers achieve higher F-scores in 118 of the 127 drugs. All cytotoxic drugs (red-coloured) have better recall and F-scores when predicted with multi-gene markers.

Figure 3 and 4 show that the test set recall of AZD8055 and AZD7762 is much higher for multi-gene markers than for single-gene markers. Figure 5 (left) plots test set recall for all the drugs to examine whether this is a general trend. This is indeed a strong trend, with 118 out of 127 drugs having a higher proportion of correctly predicted sensitive cell lines using the multi-gene markers.

We also calculated the F1-values for each compound and classifier. F1 is defined as the equally-weighted harmonic mean of precision and recall. Therefore, high F1 values highlight markers achieving both high precision and high recall in the test set. The results of this analysis are shown in Figure 5 (right). Again, most drugs exhibit much higher F1-values using multi-gene markers than single-gene markers. Interestingly, this was not the case for nine drugs (MG-132, AZD-0530, Z-LLNle-CHO, Dasatinib, A-770041, BMS-536924, Bortezomib, Thapsigargin and Nutlin-3a). All of these drugs had either TP53 or CDKN2A as their most significant marker. Both TP53 and CDKN2A are highly reoccurring mutations and thus the higher recall of the MANOVA can at least be partially attributed to the prevalence of said mutations.

**Figure 6:**
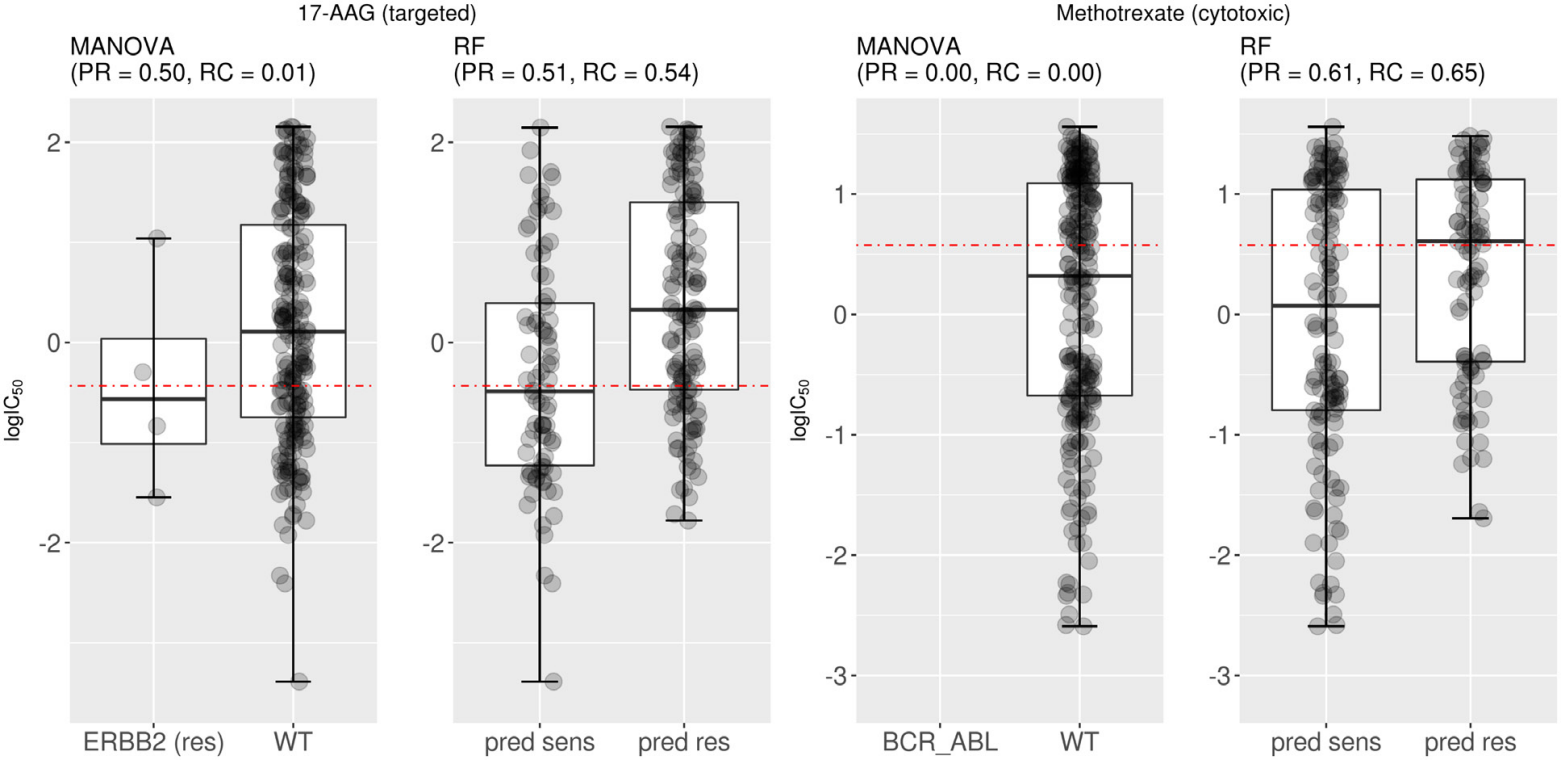
Examples of drugs having multi-gene markers with high test set recall and F-scores **(Left)** Mutated ERBB2 was the most significant single-gene marker for 17-AAG sensitivity (P=0.008) on the training set (the label “res” next to the gene indicate that ERBB2-mutant cell lines tend to be resistant to the drug). On the test set, this marker obtained a precision of 0.5 with practically no recall (RC=0.01). By contrast, the corresponding multi-gene marker has a similar level of precision with much higher recall (RC=0.54). Consequently, F-scores are also substantially higher for the multi-gene marker (F1=0.53 vs F1=0.03 of the single-gene marker). **(Right)** BCR_ABL translocation is the most significant single-gene marker for Methotrexate (P=0.0002). However, this gene fusion was not detected in any of the 234 test cell lines for this drug. For this drug, the multi-gene marker achieves a much higher test set precision and recall, which highlights that this approach is particularly useful when the single-gene marker is based on relatively rare gene mutations. In this case, F-scores are also substantially higher for the multi-gene marker (F1=0.63 vs F1=0 of the single-gene marker). Note the following abbreviations: “pred sens” (predicted sensitive) and “pred res” (predicted resistant).

We further investigated two drugs with high test set F1 by the multi-gene marker (F1=0.53 for 17-AAG and F1=0.63 for Methotrexate). Figure 6 (left) presents the test set performance of both the best single-gene marker (the mutational status of the ERBB2 gene) and the multi-gene marker for 17-AAG, also known as Tanespimycin, a HSP90 inhibitor. The recall offered by the multi-gene marker is much higher than that of the best single-gene marker for this drug (RC=0.54 vs RC=0.01), while providing similar precision (PR=0.51 vs PR=0.50). Similarly, Figure 6 (right) displays the corresponding results for Methotrexate, a cytotoxic drug primarily targeting DHFR in the G1/S phase transition of the cell cycle (DHFR plays an important role in the activation of E2F1-regulated genes during this phase). No cell line in the test set of Methotrexate harboured the genetic aberration used as response marker (BCR_ABL in this case). There are generally few tumours harbouring a given actionable mutation. In practice, this means that only a very small fraction of cancer patients can currently benefit from precision oncology [40] (i.e. single-gene markers generally providing very low recall).

### The importance of using independent test sets in biomarker discovery

**Figure 7:**
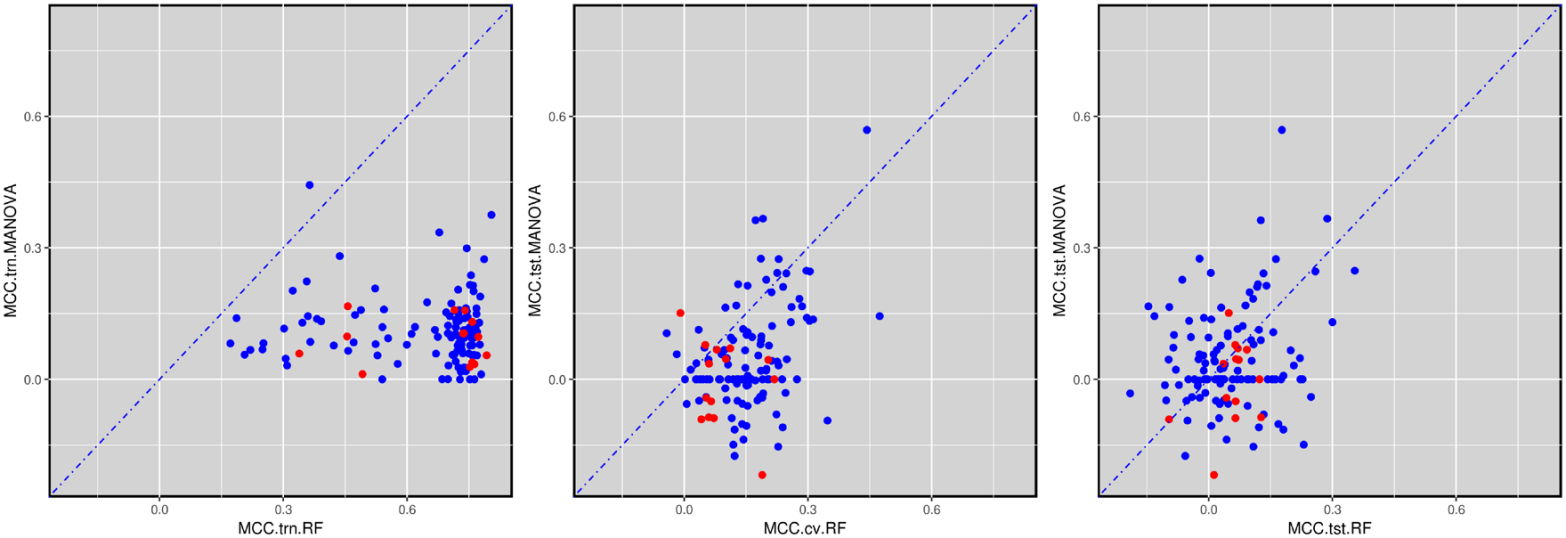
Global performance assessment of single-gene markers versus multi-gene markers across the 127 drugs **(Left)** Performance assessment based on the training data would be strongly biased towards multi-gene markers due to overfitting (99.2% of drugs are better predicted by multi-gene markers). **(Middle)** The performance of single-gene markers on the test set is compared to the 10-fold cross-validated performance of multi-gene markers using training data. The same cross-validation is also used for model selection (here, identifying the optimal RF’s m_try_ value) and hence this performance assessment is expected to be biased towards multi-gene markers (85.0% of drugs are better predicted by multi-gene markers). **(Right)** All the comparable data released after the initial GDSC release was used as a time-stamped test set, which is the most realistic form of retrospective performance assessment. 55.1% of the drugs are now better predicted by their multi-gene marker. Furthermore, 109 drugs are better predicted than random (MCC>0) with multi-gene markers, 66 of these by their multi-gene marker and the remaining 43 by their best single-gene marker. The large difference between these results and that of cross-validated results (55.1% vs 85%) shows that using the same cross-validation for both model selection and its assessment results in an overoptimistic view of predictive performance.

Training and testing on the very same data set leads to overly positive prediction results, as the model assimilates not only the signal but also the noise in the data. Such models are said to suffer from overfitting [41]. Therefore, we present a rigorous analysis on truly mutually independent training and test sets. We calculated the MCC on various data sets to investigate which markers provide better performance than randomly expected (MCC=0) [42]. As specified in the Methods section, RF is trained with each possible value of its control parameter m_try_. This leads to a family of 71 RF models per drug and that with the best cross-validated MCC is selected to predict drug response in the test set. The results are shown in Figure 7, which illustrates three ways to assess the predictive performance offered by both MANOVA’s single-gene and RF’s multi-gene markers across the 127 drugs. The plot on the left evaluates the MCC of both types of markers on training data, whereas that on the right visualise their MCC performance on test data. In addition, the middle plot shows the MCCs of single-gene markers on the test set against the MCCs of multi-gene markers on the test folds arising from the cross-validation employed for RF model selection.

By comparing the MCC values of the drugs on training set cell lines (left plot) against that on test set cell lines (rightmost plot), we can clearly see a shift in performance. Both single-gene and multi-gene markers overfit the training data, although the degree of overfitting is on average much higher in the multi-gene marker models. Note that overfitting can be quantified as the difference between the average MCC of the RF model on the training set (0.64) and its MCC on the independent test set (0.05).

A subtler form of overfitting occurs when using the cross-validated MCC from the multi-gene model (middle plot). Again, we can see that the MCC range is shifted towards the right compared to the MCC positions of the RF markers in the test set (right plot), with the average MCC being 0.15 (middle) and 0.05 (right) respectively. This discrepancy can be attributed to at least two reasons. First, both model selection and performance estimation have been carried out using the same data folds, which is known to overestimate performance [29, 43]. The most realistic performance estimation is by an independent test set (right plot in Figure 7), as these data were not used neither for training nor for model selection. Second, time-dependent batch effects may also have played a role to further strengthen this difference [32].

Despite being more overfitted, RF multi-gene markers are still able to perform slightly better than the MANOVA single-gene markers (average MCC_RF_= 0.05 compared to average MCC_MANOVA_= 0.04) on the test set data. This apparent contradiction is explained by the fact that different machine-learning algorithms exhibit different levels of robustness to overfitting, as it has been the case here. We have observed the same behaviour when applying RF to similar problems [44, 45]. Moreover, we provided an intuitive example of an analytical model robust to overfitting (see section 2 of the supplementary information of this paper [45]). When comparing the median MCCs instead of average MCCs, RF scores 0.72 on the training set and 0.04 on the test set (for MANOVA, the median value on the test set is just 0.02). These test set MCC values are very low, which shows how hard it generally is to predict cell sensitivity to drugs. On the positive side, Figure 7 (right) shows that we can already predict cell line sensitivity to some drugs with substantial accuracy.

A comparison of single-gene and multi-gene markers compared to what would be randomly expected is shown in Table 1. Although the difference in average MCC values between single- and multi-gene markers is very small, RF predicts 91 drugs with MCC values greater than 0. For the single-gene markers, this is only true for 65 drugs. 18 drugs (14.2% of the 127 analysed drugs) could not be sufficiently predicted with either marker. An additional set of 18 drugs offers some level of prediction with MANOVA (MCC>0), while RF does not manage to perform better than random. Conversely, 44 drugs have positive MCC values with RF, where MANOVA performs worse or equally to random. A total of 47 drugs have positive MCC values for both marker types. In general, of the 109 drugs that could be predicted better than random, 66 of these preferred the multi-gene markers, while in the remaining 43 drugs the univariate marker performed better. A full overview of each group can be found in Supplementary Table 2.

**Table 1:** Breakdown of the 127 drugs according to the sign of the test set MCC depending on the employed type of marker.

MCC.test	MANOVA>0	MANOVA < 0
**RF > 0**	47	44
**RF <0**	18	18

The MCC values on the test set are compared to what would be randomly expected (MCC= 0). Overall, RF predicts a higher proportion of drugs with MCC>0 (91 drugs from summing over the first row) than MANOVA (65 drugs from summing over the first column). However, 18 of the 127 drugs are not predicted by either marker better than random classification (14.2%).

We also investigated two factors that may influence the performance of the RF: test set class imbalance and training set data size. First of all, we found a statistically significant negative correlation (R, two-tailed Spearman rank-correlation test, P=9.5·10^-5^) between the performance of multi-gene classifier (MCC on the test set) and the test set class imbalance (quantified as the difference between the number of sensitive and resistant samples in a test set). Furthermore, test set performance of the RF model (MCC) was also linked to the size of such set (P=0.01, two-tailed Spearman rank-correlation test in R). This indicates that there is much room for improvement for multi-gene classifiers with growing data availability.

### About two thirds of the cytotoxic drugs are better predicted by multi-gene markers

Cytotoxic drugs often targets essential pathways, e.g. DNA replication or cellular metabolism processes, which severely impact rapidly proliferating cells such as tumour cells [46]. The cellular response to these compounds is drastically different from targeted approaches at the transcriptomics level. Here we further investigated if differences could already be found at the genomic level. As we can see from the plot (Figure 5), all of the 14 cytotoxic drugs have better recall and F-score than their corresponding single-gene markers. When we consider the MCC value on the independent test set, such as shown in Figure 6 (right), nine of the 14 cytotoxic drugs (64%) had better MCC values with multi-gene makers than with single-gene markers, while 5 had better MCC values with the MANOVA analysis. Despite only counting with 14 cytotoxic drugs, these findings do appear different from those reported by [31] at the transcriptomics level, where 12 of them were better predicted by a multi-gene marker.

## DISCUSSION

This study presents, to the best of our knowledge, the first systematic comparison of single-gene markers versus multi-gene machine-learning models of tumour sensitivity to drugs on independent test sets for genomic properties. Although a relevant analysis was included in a very recent study [8], this has several important limitations. First, LOBICO logic classifiers are inherently limited to up to four binary features [8], whereas machine-learning classifiers can employ any number and type of features. Second, there is the issue of biased model assessment due to also using the same cross-validation for model selection [29]. As our results with GDSC data confirmed (Figure 7), this practice leads to overly optimistic portraits of the actual performance of a classifier. Note that all the multi-gene markers reported in this paper were selected using data not used for their generalisation performance assessment.

Predictive biomarkers of drug sensitivity are increasingly important tools in drug development and clinical research [47, 48]. During the investigation of methods for cancer diagnosis and treatment, a vast amount of cancer genomics data is typically generated [49] and thus there is an urgent need to truly understand what biomarkers are and how to exploit the genomics features optimally [50]. Given that drug polypharmacology [51] is a strong and common event (recent estimations have unveiled that drugs have on average more than 11 molecular targets [52]), more than one gene alteration should often be influencing tumour sensitivity to a given drug. Here we have found that over half of the analysed drugs are better predicted by machine-learning models combining multiple somatic mutations than by classic univariate markers. Such *in vitro* multi-gene markers can now be further investigated *in vivo*.

Our results strongly suggest that multi-gene markers should routinely be considered in the analysis of pharmacogenomic data. We would thus like to invite researchers to apply this methodology to related biomarker discovery problems. For example, projects adopting more accurate disease models (e.g. primary tumours [53, 54], patient-derived xenografts [55] or patients [10, 56]), those exploring alternative molecular profiling data (e.g. secretive proteomics [57], epigenomics [58] or single-cell genomics [59]) or those involving drug combinations [60] could benefit greatly from implementing a similar analysis to further understand the characteristics of the generated markers. With this purpose, we are releasing our code along with step-by-step usage instructions (these files can be downloaded from http://ballester.marseille.inserm.fr/PRoncology-code.zip).

As rigorously reviewed in [40], the application of molecularly targeted cancer therapies remains marred by high failure rates and only a fraction of the responsive patients are correctly predicted to respond to these therapies. In this paper, we have highlighted some of the reasons why this is often the case, including the poor recall of univariate markers despite their sometimes high precision (Figure 4). By contrast, we found out that most of the 127 analysed drugs had multi-gene classifiers providing higher recall (Figure 5). These results underscore the importance of predictors that are not only able to provide high precision, but also high recall. High precision implies a low number of false positives and thus the model will avoid selecting cell lines that are actually resistant to the drug. High recall indicates a low number of false negatives, which means that a high proportion of cell lines sensitive to the drug are identified. As genomic markers continue to grow more popular in clinical settings, more attention needs to be paid to the recall of the predictive models that are used to identify responsive tumours as a part of a precision and recall oncology approach enabled by machine-learning modelling.

## MATERIALS AND METHODS

### GDSC data

From the Genomics of Drug Sensitivity in Cancer (GDSC) [61] (ftp://ftp.sanger.ac.uk/pub4/cancerrxgene/releases/), we downloaded the following files from the first data release: gdsc_manova_input_w1.csv and gdsc_manova_output_w1.csv. In gdsc_manova_input_w1.csv, there are 130 unique drugs as camptothecin was tested twice, drug IDs 195 and 1003, and we only kept the drug ID 1003 instance because drug ID 195 was not included in subsequent releases. Thus, effectively a panel of 130 drugs was tested against 638 cancer cell lines, leading to 47748 IC_50_ values (57.6% of all possible drug-cell pairs).

We also obtained the data from the last release of GDSC that uses the same experimental techniques to generate the pharmacogenomic data and selected genes as in the first release (gdsc_manova_input_w5.csv). This version contains 140 drug IDs, but only 139 unique drugs as AZD6482 was tested twice, drug IDs 156 and 1066 (drug ID 1066 was not present in the first release and thus we only kept drug ID 156). Hence 139 drugs were tested on 708 cell lines comprising 79,401 IC_50_ values (80.68% of all possible drug-cell pairs).

Downloaded “IC_50_” values are more precisely the natural logarithm of IC_50_ in μM units (i.e. negative values represent drug responses more potent than 1μM). We converted each of these values into their logarithm base 10 in μM units, which we denote as logIC_50_ (e.g. logIC_50_=1 corresponds to IC_50_=10μM), as in this way differences between two drug response values are directly given as orders of magnitude in the molar scale.

gdsc_manova_input_w1.csv also contains genetic mutation data for 68 cancer genes, which were selected as the most frequently mutated cancer genes [6], characterising each of the 638 cell lines. For each gene-cell pair, a ‘x::y’ description was provided by the GDSC, where ‘x’ identifies a coding variant and ‘y’ indicates copy number information from SNP6.0 data. As in Garnett et al. [6], a gene for which a mutation is not detected is considered to be wild-type (wt). A gene mutation is annotated if: a) a protein sequence variant is detected (x ≠{wt, na}) or b) a deletion/amplification is detected. The latter corresponds to a copy number alteration (CNA) different from the WT value of y=0<CNA<8. Furthermore, three translocations were considered (BCR_ABL, MLL_AFF1 and EWS_FLI1), bringing the total to 71 genomic features. For each of the gene fusions, cell lines are identified as fusion not-detected or the identified fusion is given (i.e. wt or mutated with respect to the gene fusion, respectively). The microsatellite instability (msi) status of each cell line was also determined. Further details can be found in the original publication [6].

### Time-stamped data partition to generate non-overlapping training and test sets

Both data releases have 127 drugs in common. Three drugs were only included in the first release (A-769662, Metformin and BI-D1870), whereas the subsequent release contained 12 new drugs (TGX221, OSU-03012, LAQ824, GSK-1904529A, CCT007093, EHT 1864, BMS-708163, PF-4708671, JNJ-26854165, TW 37, CCT018159 and AG-014699).

Regarding features, cell lines from both releases have been profiled for 71 common gene alterations in cancer. In addition to the three translocations and the msi status, the mutational statuses of 67 genes have been determined (i.e. those for the 68 selected genes in the first release except for the WT1 gene, which was not included in the latest release).

For each drug, there are two non-overlapping data sets. The training set contains the cell lines tested at the time of the first release (the smallest, mean and largest numbers of cell lines across drugs are 274, 366 and 506, respectively) along with their IC_50_s for the drug and 71 binary features corresponding to the presence or absence of the considered gene alterations. The test set contains the new cell lines tested for that drug and released after data in the training set (the smallest, mean and largest numbers of cell lines across drugs are 55, 203 and 358, respectively). Thus, a total of 254 data sets (one training set and one test set per drug) were generated based on the GDSC input data and analysed in this study.

### Measuring predictive performance of a classifier on a given data set

After preprocessing, pharmacogenomic data for the i^th^ drug is represented as

$$D_{i}={\left\{\left(\log IC_{50,i}^{\left(k\right)},x^{\left(k\right)}\right)\right\}}_{k=1}^{k=n_{i}}$$

Where ***x*** is a vector composed of 71 genomic features and the i^th^ drug has been tested on n_i_ cell lines. This can represent a cross-validation fold, training set or test set of any of the considered drugs.

A tumour sensitivity threshold for a given drug is required to establish which cell lines are sensitive or resistant to the drug. Sensitive cell lines are by definition those responsive to relatively small drug concentrations (i.e. cell lines obtaining low IC_50_s, or equivalently low logIC_50_s), whereas resistant cell lines are those that barely respond to such drug concentrations. As there are two classes, it makes sense to assign the 50% most sensitive cell lines to the sensitive class and the remaining 50% most resistant cell lines to the resistant class, as we would do when binarising any other continuous variable (e.g. measured size of an object to be classified as large or small). To implement this class assignment, the threshold for a given drug is defined as the median of all the logIC_50_ values (i.e. one logIC_50_ per training set cell line). Thus, positive data instances are cell lines sensitive to the drug (sensitive cell lines), which are those with logIC_50_ lower than the threshold (i.e. below the threshold). Likewise, negative data instances are cell lines resistant to the drug (resistant cell lines), which are those above the threshold.

Once the threshold is calculated, the set of all the cell lines tested with a given drug can be partitioned into four categories as defined in Figure 1, which are quantified as the numbers of true positives (TP), true negatives (TN), false positives (FP) or false negatives (FN). From this contingency table, the discrimination offered by a classifier can be summarised by the Matthews Correlation Coefficient (MCC) [62].

$$MCC=\frac{\mathrm{TP}\cdot \mathrm{TN}-\mathrm{FP}\cdot \mathrm{FN}}{\sqrt{\left(\mathrm{TP}+\mathrm{FN}\right)\cdot \left(\mathrm{FN}+\mathrm{TN}\right)\cdot \left(\mathrm{TN}+\mathrm{FP}\right)\cdot \left(\mathrm{FP}+\mathrm{TP}\right)}}$$

By the above definition of positives and negatives, MCC takes absolute values from 0 (gene mutation has absolutely no discriminative power) to 1 (gene mutation perfectly predicts whether cell lines are sensitive or resistant to the drug).

Important descriptors of the performance of biomarkers that we have used throughout the paper, are precision and recall. Precision (PR) and Recall (RC) are two classical metrics for measuring the performance of a binary classifier [63].

$$\mathrm{PR}=\frac{\mathrm{TP}}{\mathrm{TP}+\mathrm{FP}}\;\mathrm{RC}=\frac{\mathrm{TP}}{\mathrm{TP}+\mathrm{FN}}$$

Precision can be defined as the ratio of the true positives to the total number of observations that are reported as positive. Per definition, the precision is 0 if no sensitive cell line is correctly classified as being so. If no false positives are made (cell line incorrectly classified as sensitive), the precision will reach a value of 1. Recall, also known as sensitivity in binary classification, measures the fraction of successfully predicted sensitive cell lines compared to all cell lines that actually are sensitive but not necessarily found by the model. Similar to the precision, it reaches values from 0 (no sensitive cell line was correctly classified as sensitive) to 1 (no sensitive cell line was incorrectly classified as resistant).

Based on precision and recall, an alternative measure to the MCC can be derived to gain an idea of the global measure of classification performance. The F-score (F1) is the equally-weighted harmonic mean of PR and RC.

$$\mathrm{F1}=2\frac{\mathrm{PR}\cdot \mathrm{RC}}{\mathrm{PR}+\mathrm{RC}}$$

The optimal value of F1 is one indicating that both precision and recall are maximal (thus both have a value of one), whereas its worst F1 performance is at F1=0 with at least one of precision and recall being zero (when both PR and RC are zero, F1 is zero by convention).

### Single-gene markers built from the training data set

The downloaded gdsc_manova_output_w1.csv contains all the drug-gene associations with their corresponding p-values by the MANOVA test with false discovery rate of 20% with Benjamini-Hochberg correction. As such, the provided adjusted significance threshold by GDSC is 0.00840749, which we adopted. Since we only consider the 127 shared drugs and 71 common gene alterations, 8330 drug-gene associations with p-values remain. 386 of these are statistically significant after multiple hypothesis correction as calculated by Garnett et al. [6]. Each statistically significant drug-gene association was considered to be a single-gene marker of *in vitro* drug response [6]. In other words, we used these single-gene markers along with their p-values instead of carrying out this analysis again.

For each drug, the best single-gene marker for the drug was identified as the drug-gene association with the lowest p-value, as is standard procedure in biomarker discovery. This is effectively a binary classifier with a single independent variable that is built using training data alone and fixed at this model selection stage. In 14 of the 127 drugs, using the GDSC threshold, the lowest p-value among mutations was not statistically significant. However, we still used these 14 markers as otherwise multi-gene markers would immediately perform better than the single-gene approach in these drugs. Therefore, we are running a best-case scenario for single-gene markers.

Once the best single-gene marker is selected for the drug, this model is evaluated on the corresponding previously unseen test set, which is fully independent from the training data. From a machine-learning perspective, the test set simulates the availability of future data. In 26 drugs, the test set cell lines did not harbour the best mutation and thus no prediction could be made using the single-gene markers. In these cases, MCC and precision are assigned zero values.

### Multi-gene markers built from the training data set

For each of the 127 drugs, we built a Random Forest (RF) classification model [37] using the same training data sets as their best single-gene markers. Each RF model was built with 1000 trees and we defined the best value for the m_try_ control parameter for a given drug, as the value that had the lowest cross-validation root-mean-square error. All procedures were implemented in R scripts and executed using Microsoft R Open (MRO) version 3.2.5.

### Pathway information

All pathway information was obtained from Reactome [64], using database version V59.

## SUPPLEMENTARY MATERIALS TABLES









## Abbreviations

FN: number of False Negatives
FP: number of False Positives
GDSC: Genomics of Drug Sensitivity in Cancer
MCC: Matthews Correlation Coefficient
PR: Precision
RC: Recall
RF: Random Forest
TN: number of True Negatives
TP: number of True Positives
WT: Wild-Type
