# Supplementary material for: Precision and recall oncology: combining multiple gene mutations for improved identification of drug-sensitive tumours
Source: Oncotarget. 2017 Sep 15;8(57):97025–40. doi: 10.18632/oncotarget.20923 (PMC5722542; doi:10.18632/oncotarget.20923)
Supplement: Supplementary file 1 [file oncotarget-08-97025-s001.pdf]

Precision and recall oncology: combining multiple gene mutations for improved identification of drug-sensitive tumours

SUPPLEMENTARY MATERIALS

Supplementary Table 1: Results for single-gene and multi-gene markers across 127 drugs. For each drug, all obtained values from running the models can be found. These include MCC, F1, precision and recall for MANOVA (MNV) and Random Forest (RF) on training set (trn), 10-fold cross-validation (cv) and test set (tst). This information can be used to reproduce plots in this article.

See Supplementary File 1

Supplementary Table 2: Drug preferences to a given type of marker. This table shows whether the drug was best described by single-(MNV) or multi-gene (RF) markers according to MCC value. This information was summarised in Table 1.

See Supplementary File 2

Supplementary Table 3: Sensitivity threshold for each drug. The threshold is given by the median $logIC_{50}$ of training set cell lines for a given drug.

See Supplementary File 3
